# Pediatric burn resuscitation: past, present, and future

**DOI:** 10.1186/s41038-017-0091-y

**Published:** 2017-09-04

**Authors:** Kathleen S. Romanowski, Tina L. Palmieri

**Affiliations:** 1 Department of Surgery, University of Iowa Hospitals and Clinics, 200 Hawkins Drive, JCP 1500, Iowa City, IA 52242 USA; 2 Shriners Hospitals for Children Northern California, Sacramento, California USA; 3 University of California Davis, Davis, California USA

## Abstract

Burn injury is a leading cause of unintentional death and injury in children, with the majority being minor (less than 10%). However, a significant number of children sustain burns greater than 15% total body surface area (TBSA), leading to the initiation of the systemic inflammatory response syndrome. These patients require IV fluid resuscitation to prevent burn shock and death. Prompt resuscitation is critical in pediatric patients due to their small circulating blood volumes. Delays in resuscitation can result in increased complications and increased mortality. The basic principles of resuscitation are the same in adults and children, with several key differences. The unique physiologic needs of children must be adequately addressed during resuscitation to optimize outcomes. In this review, we will discuss the history of fluid resuscitation, current resuscitation practices, and future directions of resuscitation for the pediatric burn population.

## Background

Burn injury is a leading cause of unintentional death and injury in children until 14 years of age (as high as the third most common cause in children ages 5 to 9) [1, 2]. While many of these injuries are minor and can be treated as outpatients, approximately 5% are considered moderate to severe injuries and require hospitalization [3]. It is generally believed that burns larger than 15% total body surface area (TBSA) lead to the initiation of the systemic inflammatory response syndrome requiring IV fluid resuscitation to prevent burn shock and death, while smaller burns are able to be treated with oral rehydration alone [4]. Prompt resuscitation is critical in pediatric patients due to their small circulating blood volumes. Delays in resuscitation, even as short as 30 min, due to difficulty with IV access or failure to recognize size or severity of the burn can result in increased rates of complications such as acute renal failure, increased hospital length of stay, and increased mortality [5, 6]. The basic principles of resuscitation are the same in adults and children; however, children are not simply “little adults”. They have unique physiologic needs that must be adequately addressed to successfully care for burn-injured children. In this review, we will discuss the history of fluid resuscitation, current resuscitation practices, and future directions of resuscitation for the pediatric burn population.

## Review

### History of fluid resuscitation

Fluid resuscitation as a treatment for burn injury is thought to date back to the eighteenth century when Gerard van Swieten administered fluid via enema to rehydrate burn victims [7]. The idea of fluid resuscitation in burn patients gained further traction when Ludwig von Buhl made the correlation between the fluid losses in burn patients and in those with cholera and advocated for the administration of saline solution to replace losses [8]. Unfortunately, further work investigating the fluid losses and resuscitation of burn patients was not undertaken until the 1930s when Frank Underhill analyzed the composition of the fluid in blisters of burn-injured patients and found that it was similar in character to plasma [9]. The formulas that were designed for fluid resuscitation of burn-injured patients in the decade following Uphill’s work utilized plasma and based their estimates of fluid required on patient weight, and either total serum protein level or hematocrit [10–12]. The size of the burn was not a consideration in any of these formulas.

In November, 1942, Oliver Cope and Francis Moore utilized this knowledge and improved upon it as they treated the victims of the Cocoanut Grove nightclub fire. Cope and Moore recognized the relationship between the amount of fluid resuscitation required and the size of the burn. Their formula for IV fluid resuscitation used equal parts of plasma and saline and prescribed 150 ml of fluid for each 1% TBSA burn plus maintenance fluids during the first 24 h following injury [13]. Half of this fluid is given over the first 8 h, and the second half of the fluid is administered over the next 16 h (see Table 1). This pattern of fluid administration is replicated in most subsequent formulas. This was the advent of formulas that are classified as “two figure formulae” which accounted for burn-related fluid losses separate from estimations of maintenance fluid needs. The two-figure approach provides safeguards for the young, the obese, and children with large burns. The two most well known of the “two figure formulae” are the Evans formula and the Brooke formula. In 1952, the Evans formula was developed [14]. This formula utilized 2 ml/kg/%TBSA burn plus 2000 ml of maintenance fluids to replace normal losses. The Evans formula utilized one-half plasma and one-half crystalloid (normal saline). The Brooke formula was developed in 1953 by Reiss et al. and is similar to the Evans formula in that it prescribes 2 ml/kg/%TBSA burn to be administered in the first 24 h [15]. It differs in the amount of plasma that is provided, and the crystalloid used is lactated Ringer’s. The Brooke formula uses one-fourth plasma and three-fourth crystalloid.

**Table 1:** Adult formulas for burn fluid resuscitation

Formula	Crystalloid	Colloid	Glucose	Instructions for administration
Cope and Moore	75 ml/%TBSA burn oral electrolyte replacement solution	75 ml/%TBSA burn FFP	2000 ml fruit juice PO or 2000 ml 5% dextrose IV	Half over the first 8 h, half over the second 16 h
Evans	1 ml/kg/%TBSA burn of NS	1 ml/kg/%TBSA burn FFP	2000 ml 5% dextrose	Half over the first 8 h, half over the second 16 h
Brooke	1.5 ml/kg/%TBSA burn of LR	0.5 ml/kg/%TBSA burn FFP	2000 ml 5% dextrose	Half over first 8 h, half over second 16 h
Gelin	None	<30% TBSA burn: 2 ml/kg/%TBSA burn of low molecular weight dextran 30–60%TBSA: 2.5 ml/kg/%TBSA burn of low molecular weight dextran >60% 3 ml/kg/%TBSA burn of low molecular weight dextran	None	Half over the first 8 h, half the over second 16 h
Parkland	4 ml/kg/%TBSA burn of LR	None	None	Half over the first 8 h, half over the second 16 h
Revised Brooke	3 ml/kg/%TBSA burn of LR	None	None	Half over the first 8 h, half over the second 16 h

*%TBSA* percent total body surface area, *NS* normal saline, *LR* lactated Ringer’s, *FFP* fresh frozen plasma

In contrast with two figure formulae, single figure formulae do not consider the maintenance needs separate from the burn resuscitation fluids. They combine all of their fluid needs into a single formula. Gelin was the first to propose a single figure formula. He proposed three levels of fluid resuscitation. Patients with burns less than 30% TBSA received 2 ml/kg/%TBSA burn in the first 24 h, those with 30–60% TBSA received 2.5 ml/kg/%TBSA burn in the first 24 h, and those with greater than 60% TBSA received 3 ml/kg/%TBSA burn of low molecular weight dextran. Single figure formulae have become the cornerstone of adult burn fluid resuscitation. The most frequently used formula in current burn care is the Parkland formula, developed by Baxter and Shires following a series of studies on dogs [16, 17]. The Parkland formula was a departure from previous formulas because it did not use any colloid. It prescribes 4 ml/kg/%TBSA burn in the 24 h given in the manner described previously (half given in the first 8 h and the remaining half in the next 16 h). The Brooke formula was also modified to a single figure formula with a burn-injured patient receiving 3 ml/kg/%TBSA burn over 24 h of lactated Ringer’s without the addition of colloid.

### Current pediatric resuscitation practices

The initial resuscitation formulas that were developed for adult burn-injured patients were initially used in burn-injured children with “proportionally smaller quantities” [18]. This way of thinking was problematic for many reasons. Children have unique fluid needs after burn injury, and the distribution of their body surface area (BSA) is different than that of adults. Fluid resuscitation efforts for children were aided by the work of Lund and Browder who created easy-to-use diagrams for estimating burn size that took into account the differences between adults and children [19]. Unfortunately, the weight- and burn size-based formulas that have been developed and used successfully in adult patients are problematic for pediatric burn patients because they have proportionally larger BSA to mass ratios than adults which leads to under- or over-resuscitation depending on the clinical situation. In general, it has been found that the standard formulae underestimate the fluids needed. It has been suggested that pediatric patients require approximately 6 ml/kg/%TBSA burn [20, 21]. Recognition of this problem has led to the development of pediatric-specific formulas.

The principles of fluid resuscitation are similar for children and adults, but there are specific differences as well. In general, Ringer’s lactate solution should be started in patients of all age groups. Due to their limited glycogen stores, infants are at risk of hypoglycemia if a glucose source is not provided as part of their resuscitation. Their blood glucose levels should be monitored closely, and a source of 5% dextrose is provided as well. As stated earlier, the volume of fluid required per percent burn is higher in children due to their increased baseline BSA. Therefore, pediatric burn resuscitation formulas are always two figure formulae that calculate an estimated fluid resuscitation (EFR) and add maintenance fluids (MFs) with or without dextrose depending on the child’s age and size. The cutoff for using adult formulae depends on the source, but it is generally felt to be somewhere between 30 and 50 kg.

Kyle and Wallace described one of the first formulas specifically for children [22]. Their method was based on %TBSA burn, depth of injury, and determination of normal blood volume, as well as normal metabolic requirements for age and weight. The first formula that moved beyond using only patient weight was developed by Eagle in 1956 [23]. This formula uses 30 ml/%TBSA burn plus 10% of body weight in kilograms and 4000 ml/m^2^ BSA in the 48 h following injury (see Table 2). The fluid used in this formula is 5% dextrose and 0.66 normal saline with 20 g of albumin per liter. Several other formulae were developed over the next decade that used %TBSA burn, patient weight, and patient BSA to determine the initial fluid requirements [24, 25].

**Table 2:** Pediatric formulas for burn fluid resuscitation

Formula	Crystalloid	Colloid	Glucose	Instructions for administration
Eagle	30 ml/%TBSA burn + 10% weight (kg) + 4000 ml/m^2^ BSA of 0.66 normal saline	20 g of albumin per liter	5% dextrose	Administered over 48 h
Cincinnati (younger children)	4 ml/kg/%TBSA burn + 1500 ml/m^2^ total BSA of LR	12.5 g of 25% albumin per liter of crystalloid in the last 8 h of first 24 h	5% dextrose as needed	Half over the first 8 h, half over the next 16 h Composition of fluid changes every 8 h First 8 h 50 meq/l of sodium bicarbonate was added. Second 8 h was LR alone. Third 8 h adds albumin
Cincinnati (older children)	4 ml/kg/%TBSA burn + 1500 ml/m^2^ total BSA of LR	None	5% dextrose as needed	Half over the first 8 h, half over the next 16 h
Galveston	5000 ml/m^2^ BSA burn + 2000 ml/m^2^ total BSA of LR	12.5 g of 25% albumin per liter of crystalloid	5% dextrose as needed	Half over the first 8 h, half over the next 16 h

*%TBSA* percent total body surface area, *BSA* body surface area, *LR* lactated Ringer’s

In the current practice, there are two main formulas that are utilized in pediatric burn patients: the Cincinnati Formula and the Galveston Formula. Shriners Hospitals for Children in Cincinnati developed their own formula which is similar to the Parkland formula but adds in a maintenance fluid calculation based on BSA [26]. In older children, they provided lactated Ringer’s at 4 ml/kg/%TBSA burn + 1500 ml/m^2^ total BSA (1/2 of total volume over 8 h, rest of the total volume during the following 16 h). In younger children, their formula for resuscitation was much more complex. Younger children received the same 4 ml/kg/%TBSA burn + 1500 ml/m^2^ total BSA over the first 24 h, but the composition of the fluid changed every 8 h. In the first 8 h, the fluid was lactated Ringer’s with 50 mEq of sodium bicarbonate. The second 8 h was lactated Ringer’s alone, and the third 8 h was lactated Ringer’s plus 12.5 g of 25% albumin per liter. The team at Shriners Hospitals for Children in Galveston developed a formula that uses only BSA [27]. The Galveston formula provides 5000 ml/m^2^ BSA burn as a resuscitation fluid and 2000 ml/m^2^ total BSA as a maintenance fluid. As with the previously described adult formulas, half is given over the first 8 h and the remainder is given over the next 16 h. The fluid utilized in this formula is lactated Ringer’s solution with 12.5 g of 25% albumin per liter plus 5% dextrose as needed.

There has not been a head-to-head comparison of the two commonly used pediatric formulas. One group has attempted to model the use of the two different formulas over a range of patient weights and burn sizes and compare these to the physiologic norms of children, but has not examined the use of the formulas in actual practice [28]. They found that while the Cincinnati formula predicted all physiologic losses, the Galveston formula was more practical as a guideline since it allowed for more physiologic variability in their models. Beyond simply which is the “better” formula, there has also not been a study in pediatric patients that looks at predicted versus actual fluids given. This is a much needed area of study as both over- and under-resuscitation is problematic.

### Endpoints of resuscitation

While a lot of research has gone into the creation of resuscitation formulas, it is critical to remember that they are merely estimates of the amount of fluid that will be required for a given burn-injured patient. Resuscitation formulas should be used to initiate therapy. However, the IV fluid resuscitation rate should be reevaluated on an hourly basis and IV rate adjusted accordingly. Both over-and under-resuscitation are equally problematic in pediatric burn-injured patients. Under-resuscitation leads to burn shock, suboptimal tissue perfusion, end-organ failure, and death [29]. The concept of over-resuscitation or “fluid creep” was described by Dr. Pruitt in 2000 and is known to occur when burn patients are over-resuscitated with excessive amounts of fluid [30]. Despite acknowledging that over-resuscitation is occurring, it has continued to be a problem with the 24-h crystalloid volumes of three recent studies ranged from 4.6 to 6.3 ml/kg/%TBSA burn [31]. Over-resuscitation can lead to abdominal compartment syndrome [32], compartment syndrome of the limbs [33], and pulmonary edema leading to tracheostomy that might not otherwise be necessary [34].

In order to ensure that patients are not being either over- or under-resuscitated, IV fluid rates need to be adjusted based on urinary output (UO). Hourly UO continues to be the most commonly used endpoint in guiding the administration of resuscitation fluids [35]. In children weighing less than 30 kg, the UO goal is 1 ml/kg/h. In children over 30 kg, a UO of 0.5 ml/kg/h is the goal. Urine volumes less than or greater than this require adjustment in fluid resuscitation rates. Despite our reliance on this measure, the optimal hourly UO goal has never been accurately defined. “Permissive oliguria” has even been suggested as an appropriate approach [36]. Despite a focus on urine output as an endpoint, it is not the only factor in determining adequacy of resuscitation. As a resuscitation endpoint, UO is practical and works well in many cases, but it is far from perfect. The correlation between UO and measures of oxygen delivery or tissue perfusion is not strong [37]. In fact, in practitioners who do not have a lot experience, UO may be prone to misinterpretation especially in the presence of IAH or ACS where oliguria may be due to diminished renal perfusion rather than hypovolemia [38, 39]. Sheridan et al. suggest that in infants, the endpoints of resuscitation include sensorium (lightly asleep but arouses to tactile stimuli), physical examination (clear breath sounds and warm distal extremities), pulse (120–180 beats per minute), systolic blood pressure (60–80 mmHg), and urine output (1–2 ml/kg/h) [40].

In modern burn units, there are far more sophisticated methods to measure response to burn resuscitation than UO. Many of these monitoring tools are able to assess the moment-to-moment physiological state of the patient. For example, abnormal admission arterial lactate levels and base excess values correlate with the magnitude of injury and are now recognized as markers of global poor perfusion and uncompensated shock [41]. Failure of these values to correct over time predicts mortality [42–44]. There are, however, no prospective studies to support the use of lactate clearance to guide fluid resuscitation in adult or pediatric burn patients. Additionally, measures such as ScvO2 that have shown promise in guiding early goal directed therapy in the septic patient have not been examined or validated in monitoring burn resuscitation [45].

Novel methods of measuring physiologic parameters are being developed and utilized in both pediatric and adult burn patients. In adult patients, standard Parkland resuscitation was compared to resuscitation guided by invasive thermodilution (TDD) measurements in patients with greater than 20% TBSA burn [46]. This study found that patients in the TDD who directed resuscitation received significantly more fluid during the initial 24 h following injury, but there were no significant differences in preload or cardiac output parameters between the groups despite the differences in the amount of fluid they received. In the pediatric burn population, minimally invasive transpulmonary thermodilution (TPTD) has been used to measure hemodynamic parameters in 79 patients with greater than 40% TBSA burn [47]. They found that in these patients, the hyperdynamic circulation that is classically found in burn patients begins within the first week of hospitalization and continues through their entire ICU course. They did not attempt in this study to guide resuscitation using the hemodynamic parameters that they obtained. This is another area of possible study for the use of TPTD in pediatric patients.

Due to the fact that cardiac output decreases during the first 24 to 36 h after major burn injury, children with burns greater than 40% TBSA should have advanced monitoring including central venous pressures so that their response to fluids can be adequately assessed. Children receiving IV fluid rates twice that were predicted by the Parkland formula, with continued inadequate urine output, are likely to have either heart failure or other complications of over-resuscitation, including abdominal compartment syndrome or pleural effusion [48].

In general, the treatment for hypotension in pediatric burn-injured patients is fluid resuscitation. However, even proper fluid resuscitation of burn shock may not achieve complete normalization of physiologic variables due to the fact that burn injury leads to continued cellular and hormonal changes in the patient [4]. In these cases, the use of vasopressors may be warranted. One study in adults suggests that dobutamine may be a pressor of choice for burn patients as it increases the cardiac index which is shown to be low in patients who have poor outcomes [49]. Other studies suggest the use of norepinephrine for burn shock refractory to fluid resuscitation [50]. To date, there are no papers specifically on the use of pressors in the resuscitation of pediatric burn patients. While it is known that pressors are required in some burn patients, their role and choice of vasopressor remains an area in need of investigation.

### Areas of controversy and future directions

#### Colloid

The use of colloids in burn resuscitation is an area where considerable controversy exists. Plasma proteins are important in maintaining oncotic pressure to balance the outward hydrostatic pressure. The need to administer large volumes of crystalloid fluids during burn resuscitation to prevent burn shock leads to a decrease in plasma protein concentration and worsens extravascular egress of fluid and edema formation. Replacing plasma proteins using colloids (albumin, plasma, dextran, and hydroxyethyl starch) could in theory mitigate this effect. It was this theory that led to the use of colloids in the early burn resuscitation formulas such as those developed by Cope and Moore [13] and Evans et al. [14]. These early formulas utilized fresh frozen plasma (FFP), but unfortunately, this is problematic as blood products carry with them a risk of blood-borne infectious transmission and is a known risk factor for development of acute lung injury (TRALI) [51]. Due to these concerns and the fact that FFP is a limited resource, its use has largely been reserved to treat patient with active bleeding or coagulopathy.

The use of colloid at all in initial burn resuscitation was called into question by radioisotope studies conducted by Baxter and Shires [16] and Pruitt et al. [52] that demonstrated that in the early phases of resuscitation (first 24 h after injury), plasma expansion was independent of the type of fluid given because the capillary integrity is not sufficient enough to allow for colloid to influence the intravascular oncotic pressure. In fact, it has been demonstrated that early administration of colloid increases pulmonary edema. It has been demonstrated that the rate of edema formation is highest between 8 and 12 h following burn injury [53, 54]. Non-burned tissues experience a transient loss in capillary integrity and ability to sieve plasma proteins which is quickly regained. Because of this, almost all studies that have looked at the use of colloids in burn resuscitation have found reduced edema in unburned tissue but no change in the edema in the burn itself [55, 56]. The transient nature of the capillary leak has prompted some to adopt an approach where they start colloid administration during the second half of the first 24 h following burn injury. While there is evidence that indicates that adding colloid to burn resuscitation formulas can decrease volume requirements, the large multicenter randomized control studies required to fully delineate the effects of colloid have not yet been conducted.

#### Artificial colloids

There was initially a lot of interest in artificial colloids (hydroxyethyl starch (HES) and dextrans) as possible colloids to use as adjuncts to burn resuscitation in order to limit the amount of crystalloid that is given and prevent over-resuscitation and its complications. In particular, HES has been studied as an adjunct to fluid resuscitation, although none of these studies have been done in a pediatric burn population. A randomized study of 26 patients was done comparing a pure crystalloid resuscitation to one that substituted one third of the crystalloid volume with 6% HES [57]. They found that patients receiving HES required less overall fluid in the first 24 h following injury and lower C-reactive protein levels. However, when 10% HES was used in burn resuscitation, there was a trend toward increased renal failure and increased mortality although these did not reach the level of significance [58]. Despite the mixed evidence for the use of artificial colloids in burn resuscitation, the risk of impaired hemostasis, impaired renal function, and increased risk of death that was found in the use of these fluids in other critically ill populations has all but eliminated their use in the burn population [59, 60]. Given that pruritus is already a problem for burn patients, an additional concern was the severe and prolonged pruritus that is caused by the deposition of hetastarch in the skin [61].

#### Albumin

The large-scale studies needed to determine the efficacy and potential pitfalls of using albumin in children have also not been conducted at this time, but there are a few smaller studies that suggest that albumin might be beneficial in pediatric burn patients. Faraklas et al. [62] retrospectively reviewed their resuscitation protocol which utilized albumin as a rescue therapy. When patients were well above their calculated Parkland resuscitation or were beginning to show signs of complications related to edema, 1/3 of their fluid requirements were switched to 5% albumin. In order to measure the success of their resuscitation, they looked at the ratio of intake to output (I/O ratio). As patients required more fluid, their I/O ratio escalated. This ratio corrected back to the baseline levels with the addition of albumin in the study without any obvious complications of its administration. The use of albumin early (within 8–12 h of burn injury) has also been examined in children with burns larger than 15% TBSA [63]. This study found that children who received albumin early required lower crystalloid fluid volumes and had fewer fluid volume-related complications. Unfortunately, this was a very small study with only 23 patients in each group. As in adults, more research is needed in the pediatric population to determine what role albumin should play in burn resuscitation and when the ideal time for its administration is.

#### Hypertonic saline

Hypertonic saline has been suggested as a burn resuscitation fluid because it helps to correct the extracellular sodium deficit which is an important component of burn shock [64]. Hyperosmolarity helps expand plasma volume by shifting water into the intravascular space. The hyperosmolar load may also cause an osmotic diuresis which improves early urine output preventing over-resuscitation and the associated complications. The price of this shift is intracellular water depletion, but it is as yet unclear whether this intracellular water depletion is harmful to patients. Studies from the 1970s to 1980s by Monafo [65] and Moylan et al. [66] found that patients treated with hypertonic lactated saline required smaller fluid volumes to maintain adequate urine output when compared to patients treated with isotonic crystalloids. Oda et al. [67] demonstrated that the reduction in fluid volume was clinically significant as patients who received hypertonic saline had lower intra-abdominal pressures. Large-volume hypertonic saline can increase plasma sodium levels to 160 mEq/L; when this level of hypernatremia occurs, there is a decrease in urine output below 50 ml/h [68]. This level of hypernatremia is cautioned against. Therefore, in using hypertonic saline as a resuscitation fluid, it is essential to monitor serum sodium levels frequently as severe hypernatremia can lead to acute renal failure. In fact, the largest study looking at hypertonic saline was a retrospective study using historical controls which found that patients who received hypertonic saline had a fourfold increase in acute renal failure (40% vs 10.1%, *p* < 0.001) and had twice the mortality rate (53.8% vs 26.6%, *p* < 0.001) as their counterparts who received standard crystalloid resuscitation [69]. Despite the theoretical benefits and some successful trials using hypertonic lactated saline, there has been decreased enthusiasm for the use of hypertonic saline especially given the more recent data that suggests that there is an increased risk of acute kidney injury (AKI) following the administration of chloride-containing solutions in the critically ill [70]. While hypertonic saline has been used in studies of children with traumatic brain injuries, there have been no large studies looking at the use of hypertonic saline in the resuscitation of pediatric burn patients. Further research on the potential risks and benefits of hypertonic saline in the pediatric burn population must be undertaken.

#### High-dose vitamin C (ascorbic acid)

As has already been discussed, the treatment of major burn injury includes the administration of crystalloid solutions to correct hypovolemia and to restore peripheral perfusion. This post-burn volume replacement increases oxygen delivery to previously ischemic tissue, leading to ischemia-related tissue injury and the production of oxygen free radicals. Due to its antioxidant properties, there has been a significant amount of interest in the use of high-dose vitamin C in burn resuscitation to ameliorate the effects of hypovolemic and ischemic tissue damage. High-dose vitamin C is thought to protect against membrane lipid peroxidation to limit capillary leak and is a potent oxygen-derived free radical scavenger [71]. Matsuda et al. [72, 73] have conducted a number of studies in a guinea pig burn model which found that burn resuscitation with lactated Ringer’s combined with a vitamin C infusion led to reductions in the hematocrit, increased CO, decrease wound edema, and decreased fluid resuscitation volume compared with the use of lactated Ringer’s alone. These effects were seen irrespective of the depth of the wound and even when initiation of treatment was delayed by as much as 6 h post-burn injury [74]. In a 40% TBSA burn sheep model, vitamin C started within an hour of burn injury, reduced fluid requirements by 30% at 6 h and by 50% at the 48-h post-burn injury time point when compared to traditional resuscitation with lactated Ringer’s [75].

There have been two prospective human trials evaluating the use of high-dose vitamin C in burn resuscitation of adults. Mann et al. [76] conducted a blinded randomized control trial in adults with burns ≥30% TBSA that compared vitamin C at 1 g/h to normal saline which found no significant differences in net fluid intake at 24, 48, or 72 h. In an unblinded study where patients were randomized by month of admission, Tanaka et al. [77] compared resuscitation using 66 mg/kg/h ascorbic acid and lactated Ringer’s to standard resuscitation with lactated Ringer’s alone. They found that high-dose vitamin C significantly reduced 24-h resuscitation fluid volumes (45% decrease, 5.5 ml/kg vs 2.1 ml/kg) and weight gain compared with controls. Additionally, there were a decreased number of ventilator days in the treatment group as well. In addition to these prospective studies, there have been numerous retrospective studies that have used an infusion of 66 mg/kg/h of high-dose vitamin C as an adjunct to crystalloid resuscitation. These studies have found that high-dose vitamin C reduces the volume of fluid needed for resuscitation. In particular, they have found that addition of high-dose vitamin C keeps total resuscitation close to the Parkland prediction of 4 ml/kg/%TBSA burn which is up to a 30% reduction from the predicted volumes required [78, 79].

While the initial data on high-dose vitamin C infusions seems positive in its ability to reduce fluid resuscitation volumes and subsequent edema, there are a number of concerns associated with its use that have become apparent. One concern is the osmotic diuresis that occurs due to high-dose vitamin C. The fear is that patients may become dehydrated if fluids are titrated down too aggressively in light of this diuresis elevating UO values. There is some data to support this concern. When treated with high-dose vitamin C, there is an increase in UO despite a significant decrease in fluid volume [50, 51]. Additionally, high-dose vitamin C did not seem to have an effect on markers of resuscitation other than UO, such as vasopressor requirements or base deficit. Due to concerns about safety and unclear efficacy, there have yet to be studies using high-dose vitamin C as an adjunct to resuscitation in the pediatric population, although anecdotally there are some centers that use it in children. There has, however, been a small study in children looking at the effects of oral supplementation of vitamin C, vitamin E, and zinc on wound healing. They found that when vitamin C is given at 1.5 times the upper intake limit, wounds healed faster than in the untreated group [80]. The use of high-dose vitamin C in the pediatric burn population is another area that needs further study to determine if the beneficial effects seen apply to children or if the risks seen in adults outweigh the benefits.

#### Computer-aided resuscitation protocols

In burn resuscitation, it is important to remember that one is always dealing with a shock state and are often walking the line between class II and class III shock. The practitioner’s level of discomfort with a patient in the shock state and our desire to eliminate it as quickly as possible often leads to over-resuscitation and its associated complications. In an effort to deal with practitioner discomfort and standardize resuscitation as much as possible, computerized systems have been developed. Closed-loop resuscitation is a fully automated system that leads to continuous adjustment of the fluid infusion rate based on a computer-controlled algorithm. The device adjusts the infusion rate to achieve a defined physiological endpoint (in most cases, this is UO). A study by Hoskins et al. [81] compared technician-guided resuscitation with the closed-loop system. The closed-loop system resulted in significantly less variation in fluid infusion rates and UO, a significantly lower incidence of UO being under target range, and a trend toward lower total fluid volumes for resuscitation in the first 24 h.

Most physicians are not comfortable with a completely closed-loop system. This led to the development of the Computerized Decision Support System (CDSS) [82]. This system provides recommendations for fluid resuscitation to the clinician on an hourly basis, and then, the clinician is free to accept or reject those recommendations. Using this system in adult patients with burns ≥20% TBSA resulted in significant reductions in total crystalloid resuscitation volumes (over the first 48 h) and UO within the target range significantly more than their historical controls [83]. This ultimately led to shorter durations of mechanical ventilation, decreased ICU length of stay, and improved rates of survival. Kulkarni et al. [36] conducted a similar study that utilized a computerized decision support tool based on an Excel spreadsheet with a series of “if–then” logical statements. The computer support group patients required fewer escharotomies and no episodes of abdominal compartment syndrome. Patients in the computer support group did experience 1 to 2 h periods of anuria, between 6 and 10 h post-burn that did not occur in the control patients. This did not however result in episodes of early renal failure. While the use of computerized resuscitation protocols is promising, there have not been any large-scale multicenter studies, and it has yet to be tested in a pediatric burn population. Computer-aided resuscitation protocols could be especially helpful in children as they are particularly sensitive to over- and under-resuscitation.

## Conclusions

Resuscitation of burned children has improved markedly over the years. Adequate fluid resuscitation is essential to optimizing the survival of burned children. Although multiple regimens and fluids are available for resuscitation, alteration of fluid infusion rate guided by clinical endpoints is the mainstay of therapy. In the future, closed-loop resuscitation methods may improve pediatric burn outcomes.

## References

[1] Centers for Disease Control and Prevention, National Center for Injury Prevention and Control (2016). Web-based Injury Statistics Query and Reporting System (WISQARS) fatal injury data.

[2] Centers for Disease Control and Prevention, National Center for Injury Prevention and Control (2016). Web-based Injury Statistics Query and Reporting System (WISQARS) nonfatal injury data.

[3] Wolf SE, Debroy M, Herndon DN (1997). The cornerstones and directions of pediatric burn care. Pediatr Surg Int.

[4] Schulman CI, King DR (2008). Pediatric fluid resuscitation after thermal injury. J Craniofac Sur.

[5] Barrow RE, Jeschke MG, Herndon DN (2000). Early fluid resuscitation improves outcomes in severely burned children. Resuscitation.

[6] Wolf SE, Rose JK, Desai MH, Mileski JP, Barrow RE, Herndon DN (1997). Mortality determinants in massive pediatric burns. An analysis of 103 children with > or = 8% TBSA burns (> or = 70% full thickness). Ann Surg.

[7] Elgjo GI (2003). Small volume hypertonic fluid treatment of burns. J Burns Surg Wound Care.

[8] von Buhl L (1855). Epidemische cholera. Ztschr f rat Med.

[9] Underhill FP (1930). The significance of anhydremia in extensive superficial burns. JAMA.

[10] Black DA (1940). Treatment of burn shock with plasma and serum. BMJ.

[11] Elman R (1940). The therapeutic significance of plasma protein replacement in severe burns. JAMA.

[12] Lam CR (1941). Plasma therapy of burns. Ann Surg.

[13] Cope O, Moore FD (1947). The redistribution of body water and the fluid therapy of the burned patient. Ann Surg.

[14] Evans EI, Purnell OH, Robinett PW, Batchelor A, Martin M (1952). Fluid and electrolyte requirements in severe burns. Ann Surg.

[15] Reiss E, Stirman JA, Artz CP, Davis JH, Amspacher WH (1953). Fluid and electrolyte balance in burns. JAMA.

[16] Baxter CR, Shires GT (1968). Physiological response to crystalloid resuscitation of severe burns. Ann NY Acad Sci.

[17] Fakhry SM, Alexander J, Smith D, Meyer AA, Peterson HD (1995). Regional and institutional variation in burn care. J Burn Care Rehabil..

[18] Carvajal HF (1994). Fluid resuscitation of pediatric burn victims: a critical appraisal. Pediatr Nephrol.

[19] Lund CC (1944). Browder NC “The estimation of areas of burns”. Surg Gynecol Obstet.

[20] Graves TA, Cioffi WG, McManus WF, Mason AD, Pruitt BA (1988). Fluid resuscitation of infants and children with massive thermal injury. J Trauma..

[21] Merrell SW, Saffle JR, Sullivan JJ, Navar PD, Kravitz M, Warden GD (1986). Fluid resuscitation in thermally injured children. Am J Surg..

[22] Kyle MJ, Wallace AB (1951). Fluid replacement in burnt children. Br J Plast Surg.

[23] Eagle JF (1956). Parental fluid therapy of burns during the first 48 hours. NY J Med.

[24] Batchelor ADR, Kirk J, Sutherland AB (1961). Treatment of shock in the burned child. Lancet.

[25] Welch KJ, Benson CD, Mustard WT, Ravitch MM (1962). Thermal burns. Pediatric Surgery.

[26] Chung DH, Holcomb GW, Murphy JP (2009). Herndon DN. Ashcraft’s Pediatric Surgery.

[27] Carvajal HF (1980). A physiologic approach to fluid therapy in severely burned children. Surg Gynecol Obstet.

[28] Marinov Z, Kvalténi K, Koller J (1997). Fluid resuscitation in thermally injured pediatric patients. Acta Chir Plast.

[29] Chung KK, Blackbourne LH, Wolf SE, White CE, Renz EM, Cancio LC, Holcomb JB, Barillo DJ (2006). Evolution of burn resuscitation in operation Iraqi freedom. J Burn Care Res.

[30] Pruitt BA (2000). Protection from excessive resuscitation: “pushing the pendulum back.”. J Trauma.

[31] Cartotto R, Greenhalgh DG, Cancio LC. Burn state of the science: fluid resuscitation. J Burn Care Res. 2017;38(3):e596–e604. 10.1097/BCR.0000000000000541 28328669

[32] Ivy ME, Atwe NA, Palmer J, Possenti PP, Pineay M, D’Aiuto M (1994). Intra-abdominal hypertension and abdominal compartment syndrome in burn patients. J Trauma.

[33] Sheridan RL, Tompkins RG, McManus WF, Pruitt BA (1994). Intracompartmental sepsis in burn patients. J Trauma.

[34] Zak AL, Harrington DT, Barillo DJ, Lawlor DF, Shirani KZ, Goodwin CW (1999). Acute respiratory failure that complicates the resuscitation of pediatric patients with scald injury. J Burn Care Rehabil.

[35] Greenhalgh DG (2010). Burn resuscitation: the results of the ISBI/ABA survey. Burns.

[36] Kulkarni S, Harrington DT, Heffernan D, Stephen A, Connolly M, Adams C (2013). Tolerance of oliguria improves burn resuscitation. J Burn Care Res..

[37] Dries DJ, Waxman K (1991). Adequate resuscitation of burn patients may not be measured by urine output and vital signs. Crit Care Med.

[38] Saffle JR (2007). The phenomenon of “fluid creep” in acute burn resuscitation. J Burn Care Res.

[39] Cancio LC, Chavez S, Alvarado-Ortega M, Barillo DJ, Walker SC, McManus AT, et al. Predicting increased fluid requirements during the resuscitation of thermally injured patients. J Trauma. 2004;56:404–13. 10.1097/01.TA.0000075341.43956.E4 14960986

[40] Sheridan R, Remensnyder J, Prelack K, Petras L, Lydon M (1998). Treatment of the seriously burned infant. J Burn Care Rehabil.

[41] Cochran A, Edelman LS, Saffle JR, Morris SE (2007). The relationship of serum lactate and base deficit in burn patients to mortality. J Burn Care Res.

[42] Cancio LC, Galvez E, Turner CE, Kypreos NG, Parker A, Holcomb JB (2006). Base deficit and alveolar-arterial gradient during resuscitation contribute independently but modestly to the prediction of mortality after burn injury. J Burn Care Res..

[43] Kamolz LP, Andel H, Schramm W, Meissl G, Herndon DN, Frey M (2005). Lactate: early predictor of morbidity and mortality in patients with severe burns. Burns..

[44] Andel D, Kamolz LE, Roka J, Schramm W, Zimpfer M, Frey M (2007). Base deficit and lactate: early predictors of morbidity and mortality in patients with burns. Burns..

[45] Rivers E, Nguyen B, Havstad S (2001). Early goal directed therapy in the treatment of severe sepsis and septic shock. N Engl J Med.

[46] Holm C, Mayr M, Tgeler J, Hörbrand F, Henckel von Donnersmarck G, Mühlbauer W, et al. A clinical randomized study on the effects of invasive monitoring on burn shock resuscitation. Burns. 2004;30(8):798–807. 10.1016/j.burns.2004.06.016 15555792

[47] Branski LK, Herndon DN, Byrd JF, Kinsky MP, Lee JO, Fagan SP (2011). Transpulmonary thermodilution for hemodynamic measurements in severely burned children. Crit Care.

[48] Palmieri TL (2016). Pediatric burn resuscitation. Crit Care Clin.

[49] Bernard F, Gueugniaud PY, Bertin-Maghit M, Bouchard C, Vilasco B, Petit P (1994). Prognostic significance of early cardiac index measurements in severely burned patients. Burns.

[50] Venet F, Plassais J, Textoris J, Cazalis MA, Pachot A, Bertin-Maghit M (2015). Low-dose hydrocortisone reduces norepinephrine duration in severe burn patients: a randomized clinical trial. Crit Care..

[51] Gajoc O, Rana R, Mendez JL, Rickman OB, Lymp JF, Hubmayr RD (2004). Acute lung injury after blood transfusion in mechanically ventilated patients. Transfusion..

[52] Pruitt BA, Mason AD, Moncrief JA (1971). Hemodynamic changes in the early postburn patient: the influence of fluid administration and of a vasodilator (hydralazine). J Trauma.

[53] Carvajal HF, Parks DH (1988). Optimal composition of burn resuscitation fluids. Crit Care Med.

[54] Demling RH (2005). The burn edema process: current concepts. J Burn Care Rehabil.

[55] Demling RH, Kramer GC, Gunther R, Nerlich M. Effect of nonprotein colloid on postburn edema formation in soft tissues and lung. Surgery. 1984;95:593–602. 6200946

[56] Guha SC, Kinsky MP, Button B, Herndon DN, Traber LD, Traber DL (1996). Burn resuscitation: crystalloid versus colloid versus hypertonic saline hyperoncotic colloid in sheep. Crit Care Med..

[57] Vlachou E, Gosling P, Moiemen NS (2010). Hydrixyethylstarch supplementation in burn resuscitation—a prospective randomized control trial. Burns.

[58] Bechir M, Puhan MA, Neff SB (2010). Early fluid resuscitation with hyperoncotic hydroxyethyl startch 200/0.5 (10%) in severe burn injury. Crit Care.

[59] Cope JT, Banks D, Mauney MC, Lucktong T, Shockey KS, Kron IL, et al. Intraoperative hetastarch infusion impairs hemostasis after cardiac operations. Ann Thorac Surg. 1997;63:78–82. 10.1016/s0003-4975(96)01071-5 8993245

[60] Lissauer ME, Kramer ME, Johnson SB, Scalea TM, Johnson SB (2011). Association of 6% hetastarch with adverse outcomes in critically ill trauma patients. Am J Surg.

[61] Groeneveld JAB (2000). Albumin and artificial colloids in fluid management: where does the clinical evidence of their utility stand?. Crit Care.

[62] Faraklas I, Lam U, Cochran A, Stoddard G, Saffle J (2010). Colloid normalizes resuscitation ratio in pediatric burns. J Burn Care Res.

[63] Müller Dittrich MH, de Brunow Carvalho W, Lavado EL (2016). Evaluation of the “early” use of albumin in children with extensive burns: a randomized controlled trial. Ped Crit Care Med.

[64] Moyer CA, Margraf HW, Monafo WW (1965). Burn shock and extravascular sodium deficiency—treatment with Ringer’s solution with lactate. Arch Surg.

[65] Monafo WW (1970). The treatment of burn shock by the intravenous and oral administration of hypertonic lactated saline solution. J Trauma.

[66] Moylan JA, Reckler JM, Mason AD (1973). Resuscitation with hypertonic lactate saline in thermal injury. Am J Surg.

[67] Oda J, Ueyama M, Yanashita K, Inoue T, Noborio M, Ode Y (2006). Hypertonic lactated saline resuscitation reduces the risk of abdominal compartment syndrome in severely burned patients. J Trauma..

[68] Shimazaki S, Yoshioka T, Tanaka N, Sugimoto T, Onji Y. Body fluid changes during hypertonic lactated saline solution therapy for burn shock. J Trauma. 1977;17:38–43. 10.1097/00005373-197701000-00005 833904

[69] Huang PP, Sticky FS, Dimick AR, Treat RC, Bessey PQ, Rue LW (1995). Hypertonic sodium resuscitation is associated with renal failure and death. Ann Surg..

[70] Yunos NM, Bellomo R, Hegarty C, Story D, Ho L, Bailey M (2012). Association between a chloride-liberal vs. chloride-restrictive intravenous fluid administration strategy and kidney injury in critically ill adults. JAMA.

[71] Horton JW (2003). Free radicals and lipid peroxidation mediated injury in burn trauma: the role of antioxidant therapy. Toxicology.

[72] Matsuda T, Tanaka H, Williams S, Hanumadass M, Abcarian H, Reyes H (1991). Reduced fluid volume requirement for resuscitation of third degree burns with high-dose vitamin C. J Burn Care Rehabil..

[73] Matsuda T, Tanaka H, Shimazaki S, Matsuda H, Abcarian H, Reyes H (1992). High-dose vitamin C therapy for extensive deep dermal burns. Burns..

[74] Sakurai M, Tanaka H, Matsuda T, Goya T, Shimazaki S, Matsuda H (1997). Reduced resuscitation fluid volume for second-degree burns with delayed initiation of vitamin C therapy (beginning 6 hours after injury). J Surg Res..

[75] Dubick MA, Williams C, Elgjo GI, Kramer GC (2005). High-dose vitamin C infusion reduces fluid requirements in the resuscitation of burn-injured sheep. Shock.

[76] Mann R, Foster K, Kemalyan N, Gibran NS, Engrav LH, Heimbach DM (1997). Intravenous vitamin C in clinical burn resuscitation. J Burn Care Rehabil.

[77] Tanaka H, Matsuda T, Miyagantani Y, Yukioka T, Matsuda H, Shimazaki S (2000). Reduction of resuscitation fluid volumes in severely burned patients using ascorbic acid administration: a randomized prospective study. Arch Surg..

[78] Schulman CI, Cofnas P, Manning R, Quintana O, Varas R, Pizano LR (2011). Use of high dose vitamin C in burn resuscitation. J Burn Care Res..

[79] Lentz CW, Huelskamp S, Reid D (2014). Adjuvant high dose ascorbic acid reduces both the volume of burn resuscitation fluids and the time to complete resuscitation in burn shock. J Burn Care Res.

[80] Barbosa E, Faintuch J, Machado Moreira EA, da Silva VR G, Lopes Pereima MJ, Martins Fagundes RL, Filho DW (2009). Supplementation of vitamin E, vitamin C, and zinc attenuates oxidative stress in burned children: a randomized, double-blind, placebo-controlled pilot study. J Burn Care Res.

[81] Hoskins SL, Elgjo GI, Lu J, Ying H, Grady JJ, Herndon DN, et al. Closed-loop resuscitation of burn shock. J Burn Care Res. 2006;27:377–85. 10.1097/01.BCR.0000216512.30415.78 16679909

[82] Salinas J, Drew G, Gallagher J, Cancio LC, Wolf SE, Wade CE (2008). Closed-loop and decision assist resuscitation of burn patients. J Trauma..

[83] Salinas J, Chung KK, Mann EA, Cancio LC, Kramer GC, Serio-Melvin ML (2011). Computerized decision support system improves fluid resuscitation following severe burns: an original study. Crit Care Med..

